# Transient painful vision loss due to hypotony after an intravitreal injection in a previously vitrectomized eye

**DOI:** 10.1016/j.ajoc.2022.101487

**Published:** 2022-03-10

**Authors:** Amer F. Alsoudi, Zeeshan Haq, Jay M. Stewart

**Affiliations:** a University of California, San Francisco, Department of Ophthalmology, San Francisco, CA, USA; b Zuckerberg San Francisco General Hospital and Trauma Center, Department of Ophthalmology, San Francisco, CA, USA

**Keywords:** Scleral wound leak, Hypotony, Intravitreal injection

## 1 Case report

A 70-year-old woman presented with persistent left eye blurry vision and soreness two days after an intravitreal injection (IVI). She had received her 27th injection of aflibercept with a 30-gauge needle in the superotemporal quadrant as treatment for cystoid macular edema due to a branch retinal vein occlusion. Her left eye history was also notable for *Fusarium* endophthalmitis with subsequent retinal detachment requiring vitrectomy, inferior retinectomy, and inferior scleral buckling four years prior to presentation. Of note, her endophthalmitis consisted of a relatively low-grade presentation, with a small hypopyon and vitritis, with normal intraocular pressure (IOP) and no chorioretinal folds throughout her course.

**Fig. 1:**
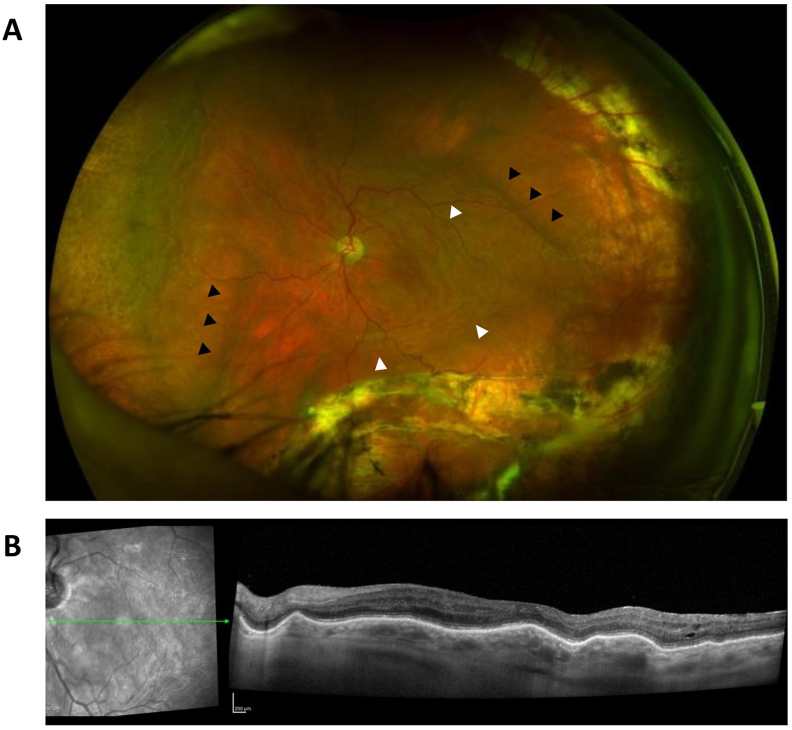
A: Fundus photograph. B: SD-OCT image. A, Left eye demonstrating diffuse small (white arrowheads) and large (black arrowheads) chorioretinal folds and vascular tortuosity in the posterior pole. B, Spectral domain optical coherence tomography image of the macula demonstrating chorioretinal undulations and trace intraretinal fluid.

Visual acuity was 20/20 OD and 20/400 OS (baseline 20/100 OS). Intraocular pressure (IOP) was 20 mm Hg OD and undetectable OS (compared to her baseline IOP of 14 mm Hg in that eye). Slit lamp examination OS showed Seidel negativity at the IVI site. No conjunctival or scleral injection was seen. The anterior chamber was deep and quiet. Indirect ophthalmoscopy OS showed diffuse folds in the posterior pole with otherwise stable findings (Fig. 1A). No vitreous opacities or haze were seen. Autofluorescence showed hyper-autofluorescent linear bands in a circumferential distribution. Optical coherence tomography (OCT) imaging of the macula showed stable intraretinal fluid with new chorioretinal undulations (Fig. 1B).

The patient was diagnosed with acute hypotony due to a sclerotomy wound leak. Topical atropine was started, and the patient reported resolution of her symptoms within 24 h. She was next examined one month later, at which point her left eye VA, IOP, clinical appearance, and OCT findings had returned to baseline. She underwent repeat IVI treatment at an adjacent location to the prior injection; the procedure was uneventful, but one day later the patient called to report the sudden onset of the same symptoms as before, namely pain and distorted vision. She was not examined but was instructed to use topical atropine again. One month later, when she was seen next, she reported that her symptoms had resolved almost immediately after starting the atropine. At that visit and subsequently, injections were performed in the inferonasal quadrant, and she experienced no further episodes of hypotony with 15 injections over a two-year follow-up period.

## 2 Discussion

Reports of vision loss in patients with an unsealed sclerotomy site after IVI with a 30-gauge needle have been published.^1^, ^2^, ^3^ Among these reports, a quarter of patients were symptomatic at initial presentation, half of patients were noted to have hypotony on post-operative day 1, and none of the reports included OCT imaging findings. The authors speculate that the absence of vitreous incarceration at the injection site increases the risk of wound leak in patients with a history of a prior vitrectomy. In addition, the development of compromised scleral integrity has been reported in the setting of repeated IVIs at the same location.^3^ Both of the aforementioned risk factors were present in the current case.

As illustrated in this case, conservative management of wound leaks after IVI is a reasonable first choice. However, sutured closure of the wound may be necessary for persistent wound leaks.^1^^,^^2^ In patients with suspected risk factors for this complication, preventative measures such as varying the injection site or adopting an angled entry can be considered.

## 3 Conclusion

Patients with a history of vitrectomy and multiple IVIs at the same location can develop a wound leak complicated by symptomatic hypotony.

## Consent statement

Patient consent was obtained by patient in writing.

## Conflict of interest disclosures

All authors have no disclosures to report.
